# A genetic algorithm-based boolean delay model of intracellular signal transduction in inflammation

**DOI:** 10.1186/1471-2105-12-S1-S17

**Published:** 2011-02-15

**Authors:** Chu Chun Kang, Yung Jen Chuang, Kai Che Tung, Chun Cheih Chao, Chuan Yi Tang, Shih Chi Peng, David Shan Hill Wong

**Affiliations:** 1 Department of Computer Science, National Tsing Hua University, Hsinchu, 30013, Taiwan, ROC; 2 Department of Chemical Engineering, National Tsing Hua University, Hsinchu, 30013, Taiwan, ROC; 3 Institute of Bioinformatics and Structural Biology, National Tsing Hua University, Hsinchu, 30013, Taiwan, ROC; 4 Department of Computer Science and Information Engineering, Providence University, Taichung, 43301 Taiwan, ROC

## Abstract

**Background:**

Signal transduction is the major mechanism through which cells transmit external stimuli to evoke intracellular biochemical responses. Understanding relationship between external stimuli and corresponding cellular responses, as well as the subsequent effects on downstream genes, is a major challenge in systems biology. Thus, a systematic approach to integrate experimental data and qualitative knowledge to identify the physiological consequences of environmental stimuli is needed.

**Results:**

In present study, we employed a genetic algorithm-based Boolean model to represent NF-κB signaling pathway. We were able to capture feedback and crosstalk characteristics to enhance our understanding on the acute and chronic inflammatory response. Key network components affecting the response dynamics were identified.

**Conclusions:**

We designed an effective algorithm to elucidate the process of immune response using comprehensive knowledge about network structure and limited experimental data on dynamic responses. This approach can potentially be implemented for large-scale analysis on cellular processes and organism behaviors.

## Background

Resolving the complex cellular signal transduction is a grand challenge in systems biology. Signal transduction involves cascade of protein-protein interaction and complex feedback loops [1] across proteomic and genomic levels. Models of the dynamics of the combined regulatory networks provide in-depth analysis temporal characteristics of targeted biological process. Furthermore, *in silico* knockout experiments by these models could help biologists to prioritize target genes of interest and reduce time and cost of real experiments.

Types of dynamic network models include kinetic models [2,3], hidden Markov models [4], and logic-based models [5,6]. Kinetic models based on differential equations have been used to elaborate dynamics on numerous systems [7]. However, they need detailed information about network structure, reaction mechanism and the respective kinetic parameters; which, unfortunately, are not easily obtainable. Hidden Markov model (HMM) is a statistical model in acyclic pathway [8]. The state is hidden, but the outcome dependent on the state is visible. Hence, HMMs are usually used to model known results with unknown process mechanism. Boolean network is a qualitative logic-based model that was introduced in the 1960s [9]. In the past few decades, scientists have frequently used Boolean network to model gene regulatory networks (GRN), apoptosis, metabolic network, immune response and signaling pathways [5,10-12]. Since logic-based or qualitative knowledge of interaction is abundant, the network structure can be easily established. Moreover, only minimal information is required to describe the dynamics of Boolean transfer function, they can be obtained using limited experimental data. Therefore Boolean model is an effective and extendable way of modeling the dynamics of signal transduction.

The transcription factor NF-κB controls various inflammation mediators to orchestra interwoven cellular responses to inflammatory stimuli such as TNF, IL-1 and TLR4 etc. In this study, Boolean model with time-delay was used to described the NF-κB signaling. The objective is to integrate qualitative information on network interactions from published datasets and dynamic response data in literatures to reveal the regulatory mechanism of infection and inflammation.

## Results and discussion

### Model

**Figure 1:**
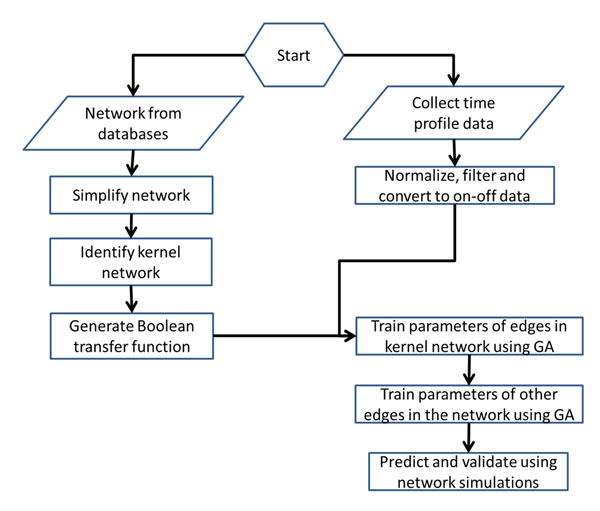
**The workflow of our strategy.** The flowchart of developing our model: First collect network structure information and experimental time profile data. Simplify the network, identify the kernel and create Boolean transfer functions for the simplified network. Experimental data are normalized, filter and converted to binary form. The parameters of the kernel pathways are trained first using genetic algorithm. Parameters of remainin pathway are then determined.

**Figure 2:**
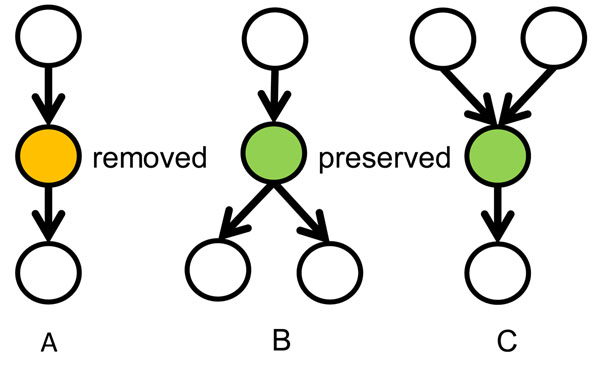
**The network simplification procedure.** In A, the orange node, which has only one input and one output is removed. In B, C, The green nodes, which has more than one input or more than one output are preserved.

**Figure 3:**
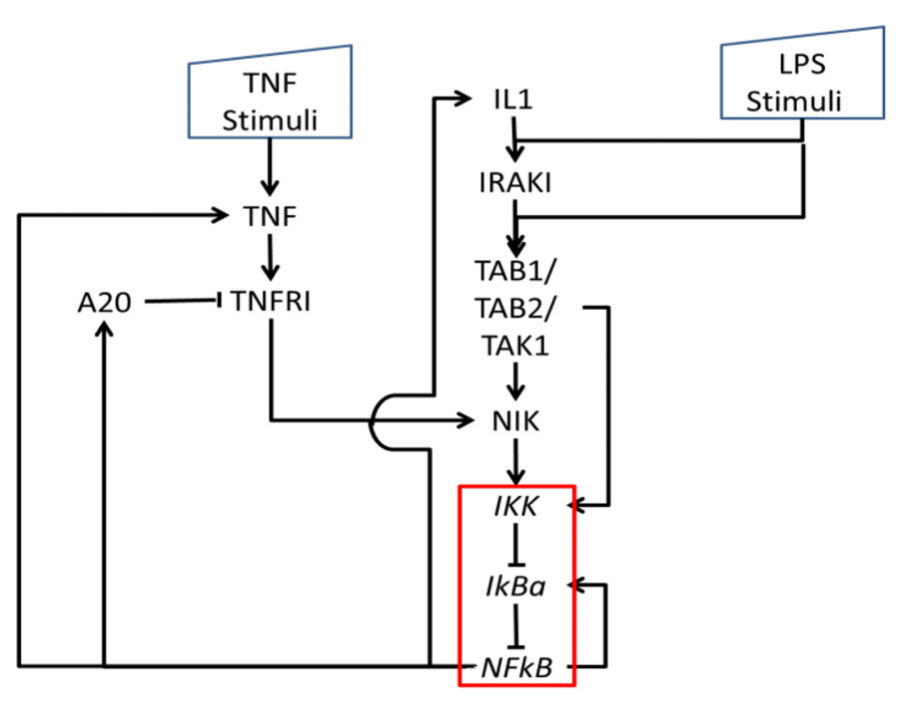
**LPS/IL1/TNF → NF-κB network after simplification.** LPS/IL1/TNF induced network after simplification. In the network, TNF, IL-1 and LPS are inputs. A-20, IL-1 and TNF are three key feedbacks. NF-κB, IKK and IκBα are observable outputs which can be detect from experiment. An edge between nodes indicates interaction. *Arrow* implies activation *blunted line* denotes inhibition. Within the red box is the kernel pathway of between IKK, IkBa and NFkB.

Figure 1 shows the workflow of building our Boolean model with time delay. First, we generated the Boolean transfer function of our model. Oda et al [16] provided a comprehensive intracellular molecular interaction map. The information was integrated with pathways from KEGG database to obtain a comprehensive network. We focus on the network between three receptors: Interleukin 1 (IL-1), Toll-like receptor 4 (TLR4) and Tumor necrosis factor (TNF) and observable outputs are IKK, IkBa, TNFa. External stimuli considered are TNF and LPS. The network contains a lot of sequential relations which can be compressed using the method shown in Figure 2. In the compression, an intermediate node with only one input and one output is removed; Any branching or meeting nodes are preserved. Figure 3 illustrates the simplified cascade and feedback of signals. There are three feedback routes involving TNF, A20, and IL1. A kernel pathway involving IKK, IkBa and TNFa can be identified through which all the signals have to go through.

Boolean transfer functions were used for each edge. Each transfer function has two dynamic parameters. The delayed activation θ denotes the duration that the input to a node must turned on before the reception node is turned on. The sustained response r is the time that the output of a node can be sustained once it is turned on. These parameters were obtained by fitting a training data set published in [2,17,18] using GA.

Even with a simplified network and limited number of dynamic parameters, convergence to a set of reasonable parameters was not easy. In order to improve the modeling process, we trained model parameters in kernel pathway first with parameters of the rest of the edges set equal to 1, using wild type data containing measurements of both IKK and NFkB measurements. When kernel’s parameters were decided, the parameters of the remaining edges were determined by adding additional data involving A20 knockout, stimulus of various strengths and measurements of either IKK or NFkB only.

**Figure 4:**
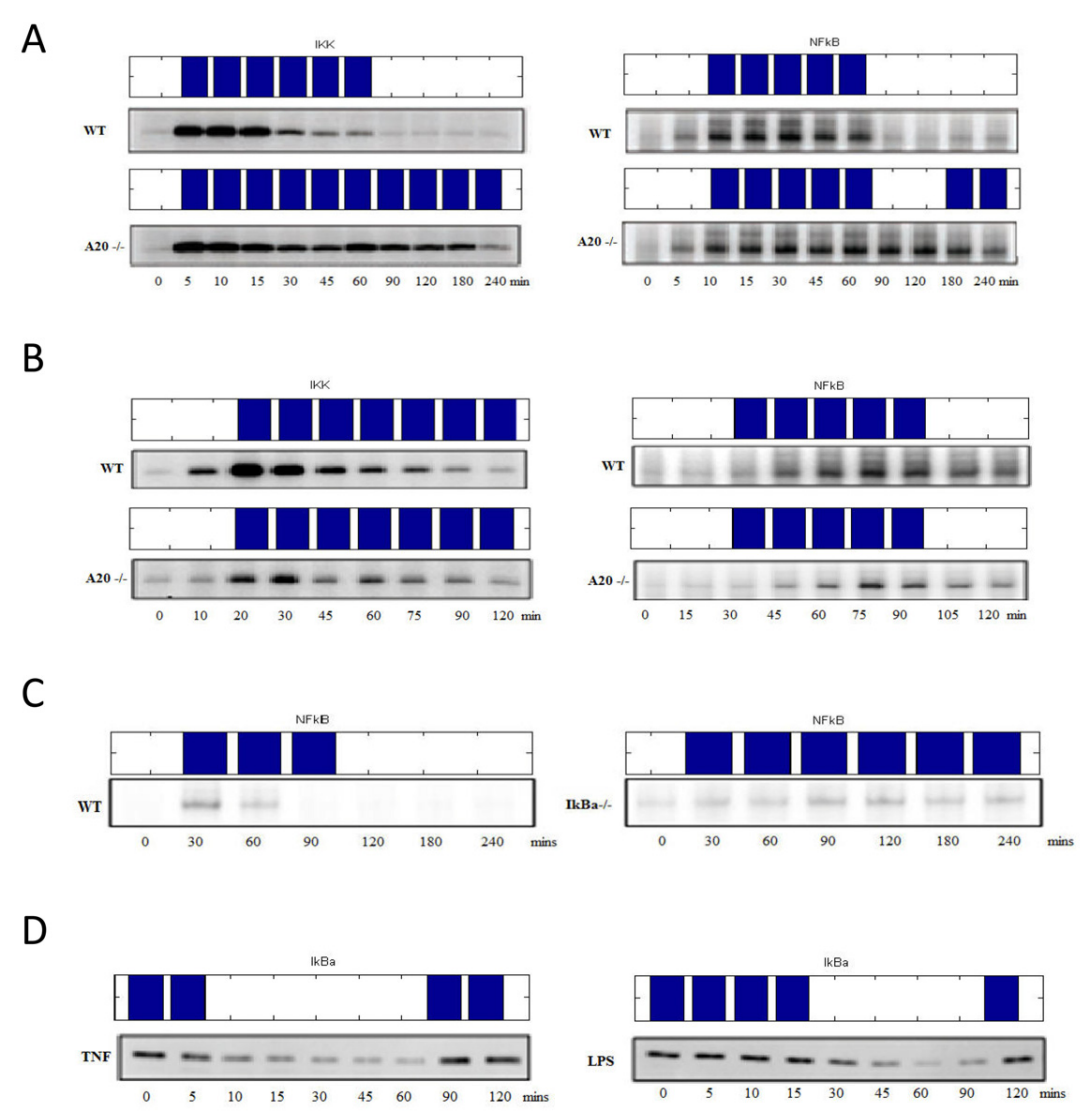
**The pattern of model simulation compare with experimental data.** Simulations profile of our model (Upper boxes) and coresponding original data [2,17,18]. Blue bars represent on and white space means off, respectively. X-axis is the time course (minutes) and y-axis means the activities of target in our model in distinct conditions: (A) IKK (left) and NFkB’s (right) activities induced by TNF 45 minutes treatment with wild type and A20 mutant. (B) IKK (left) and NFkB’s (right) activities with wild type and A20 knockout condition of LPS 45 minutes treatment. (C) NFkB’s activities induced by transient 15 minutes IL-1 in wild type and IκBα mutant. (D) IkBa’s activities with TNF (left) and LPS (right) 45 minutes stimuli.

Figure 4 shows the comparison between our model outcome and the experimental data in the learning sets. The upper boxes are each active pattern with specific treatment (the description was written on the graph) and compared with real data (the western blot data under corresponded box). The MSE value between our model and data is 0.0919.

### Dynamics implications

The parameters obtained for the Boolean model were shown in Table 1. The delay of transcription factor NFkB induced components, such as A20, IkBa, IL1 and TNF are longer than other reactions in Table 1. It reveals transcription costs more time than phosphorylation. TNF related pathways will response immediately in our model. In contrast, LPS costs longer delay time to start immune response. The activation of IKK by LPS through TAB1/TAB2/TAK1 is much slower than activation through NIK by TNF.

**Table 1:** The model obtained by our approach

Component	Boolean transfer function
IRAK1(t)	= 5*IL1(t-1) **OR** 3*LPS(t-87)
TAB1/TAB2/TAK1(t)	= IRAK1(t-13) **OR** LPS(t-125)
NIK(t)	= TAB1/TAB2/TAK1(t-25) **OR** TNFR1(t-1)
IKK(t)	= TAB1/TAB2/TAK1(t-100) **OR** NIK(t-1)
IkBa(t)	= **NOT** 2* IKK(t-3) **OR** -62* NFkB(t-96)
NFkB(t)	= **NOT** IkBa(t-1)
A20(t)	= 7*NFkB(t-107)
IL1(t)	= -62*NFkB(t-373)
TNF(t)	= -62*NFkB(t-69)
TNFR1(t)	= 4*TNF(t-1) **AND NOT** A20(t-10)

Each component has a Boolean transfer function that contains logic gates and two type of parameters: delay activation (inside bracket) and sustained response (before * sign). Each time step corresponds to 30 second in real time

### Negative regulator

**Figure 5:**
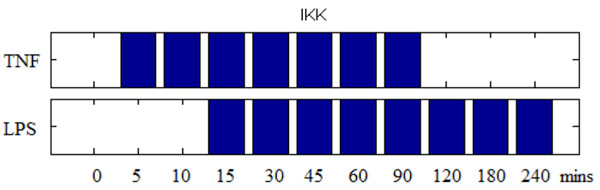
**Effect of IκBa deletion**. Simulated IKK’s active pattern obtained by 45 min stimuli of TNF and LPS and IkBa knockout.

Because NFkB regulates pro-inflammatory cytokines, such as IL1, TNF, and negative regulator A20 [19], these effectors can generate positive and negative feedback control. In our model, IL1 and TNF were positive feedback. Excessive cytokine production is harmful to the host due to its effects on blood circulation system. Thus, “Endotoxin tolerance” is a critical negative feedback mechanism to protect host from endotoxic shock [20]. In our model, negative regulator A20 and IkBa could suppress NFkB’s activity. Specifically, NFkB induced IkBa (nuclear factor of kappa light polypeptide gene enhancer in B-cells inhibitor, alpha) will supress NFkB activity in a self-regulatory cycle. As shown in Figure 5, inflammation response will be prolonged by IkBa deletion in TNF and LPS stimulation.

Zinc finger protein A20, also called TNFAIT3 (Tumor necrosis factor, alpha-induced protein 3), can also produce RIP- or TRAF2- mediated signal to indirectly block the NFkB activity. We have learned from the literatures that the negative regulator A20 blocked the NFkB activation while protecting the host cell from TNF-mediated apoptosis. To mimic an A20 knockout assay done in the wet-bench experiments, we set the output of the A20 nod to 0 and keep it in off state during simulation as the deletion. The resulting signaling pattern of wild type and A20 mutation in our model were shown in Figure 4. TNF induced IKK(left side of Figure 4.A) active from 5 min to 60 min in wild type and persist with A20 deletion. NF-κB(right side of Figure 4.A) caused secondary activation when A20 is knocked out. For LPS-induced IKK (left side of Figure 4.B) and NFkB (right side of Figure 4.B), there is no difference between active patterns obtained with wild-type and A20 deletion. This is because in a LPS induced response, the secondary TNF response will be triggered after the transcription of NFkB. Hence A20 is apparently a key component in TNF-induced pathway but with no significant influence in LPS-induced pathway.

### Clinical implication

**Figure 6:**
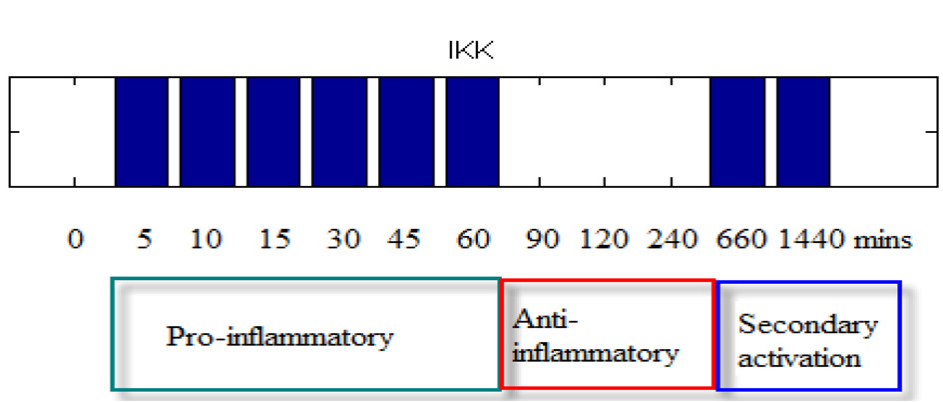
**Pro-inflammatory and anti-inflammatory period.** Simulated IKK’s active pattern obtained by continuous TNF stimulation. The time profile can be separated as pro-inflammation and anti-inflammation period. Due to continuous TNF infection, it will cause secondary activation.

Figure 4.A and Figure 6 can help us understand acute and chronic inflammatory response. In acute infection, IKK will active for short span of time to initiate the immune response and return the system to steady state quickly. On the other hand, under chronic inflammatory response (shown in Figure 6), IKK has an oscillatory profile that can generate greater cytokines production to protect the host cell. With our model, it is hence possible to separate the time course into two phases: the pro-inflammatory and anti-inflammatory phases. As mentioned before, NFkB is a key regulator for inflammation. It up-regulates pro-inflammatory cytokines such as TNF and IL-1 as well as trigger negative regulator such as A20 and IkBa to suppress the IKK activities. Over a short span of 60 minutes, IKK’s activity will decrease by the repression of IkBa, this process can thus be defined as the anti-inflammatory phase. Further, the time profile of TNF-induced IKK can cause secondary activation around 6 hour.

## Conclusions

In this work, a dynamic Boolean model was generated by integrating and comprehensive qualitative knowledge about network structure and fitting a minimal amount of dynamic response data. The model is capable of capturing feedback and crosstalk dynamics between diverse signaling pathways. Using this model, mechanisms of and factors affecting periodic pro-inflammatory and anti-inflammatory responses can be elucidated.

The proposed approach integrated intracellular and intercellular process. Hence it is possible for us to use this approach to develop system models for host defense against the shock from environmental or pathogen stimuli and predict the inflammatory response. Such a model will potentially be able to provide insight to a feedback treatment scheme for clinical therapy.

## Methods

### Boolean transfer function

The Boolean model with time delay can be described mathematically by the graph *G*={*V,E*}, in which *V* = {*x*_1_ ⋯ *x_N_*} is a set of nodes and *E*={*e_ij_*} is a set of edges, with *e_ij_* equals 1 if there is an linking edge starting from the j^th^ to the i^th^ node and *e_ij_* equals 0 otherwise [21-23]. The transfer function that determine activation of the node *x_i_* at time *t* is given by:

:

The effect of accumulated activation is given by

In other words, the activation of the i^th^ node by j^th^ node is on only if the j^th^ node has been on continuously for a period of *θ_ij_*. The effect of sustained and delayed response can be described by the following pseudocode:

when *h*({*x*_1_(*t*)}, *θ_i1_*) = 1

if *r_ij_* ≥ 1

*τ_ij_* = *τ_ij_* + *r_ij_* - 1

*g_ij_* = 1

else

*τ_ij_* = *τ_ij_* + 1

if *τ_ij_* ≥ |*r_ij_*|

*g_ij_* = 1

*τ_ij_* = *τ_ij_* - 1

end

end

Thus if *r_ij_* ≥ 1, the activation of *x_i_* by *x_j_* will be sustained; alternatively the activation of *x_i_* by *x_j_* will be delayed.

**Figure 7:**
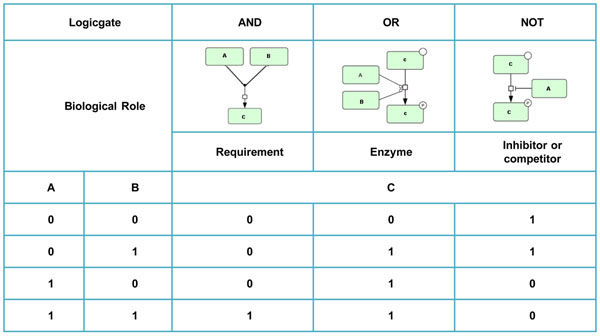
**Logical gates to biological processes.** Boolean network include combinations of logic operator (AND, OR, NOT) were developed from the knowledge of components directly upstream of each target node in the network. The logic gate also called transfer function which modified with information from the literature. We use OR operator when either of upstream nodes could activate the target component. AND operator is for synergy, which means two or more upstream nodes are necessary to activate the target component. In the other case, the NOT operator represents inhibition or competition.

The function f_1_ is a series of logical gates connecting input nodes to output nodes. The relations of these logical gates to biological processes are shown in figure 7.

### Data processing

**Figure 8:**
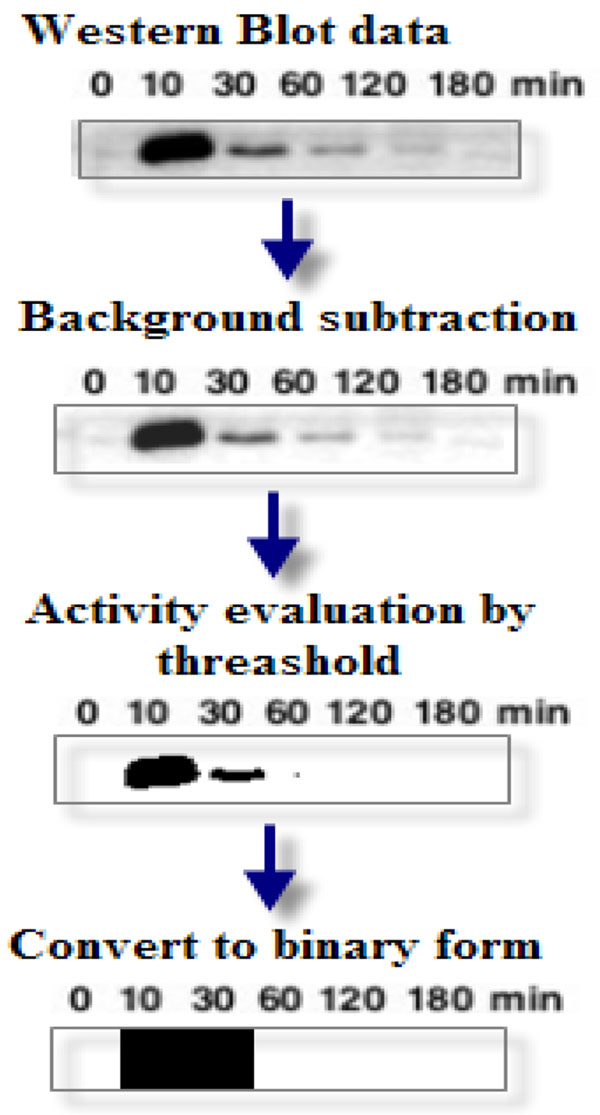
**Experimental data processing normalization**. The data processing by ImageJ software. The original data is from [2]. We subtracted background and separated profile bipartitely by Max entropy threshold approach. The binary data profile comes after data processing.

To generate on-off response of the observed nodes, we collected the Western blot experimental data in [2,17,18] and employed processing steps in ImageJ as shown in Figure 8. First the background is subtracted. Then the maximum entropy threshold approach was used to filter the data. Finally, a binary data profile is obtained.

### Model fitting by genetic algorithm

**Figure 9:**
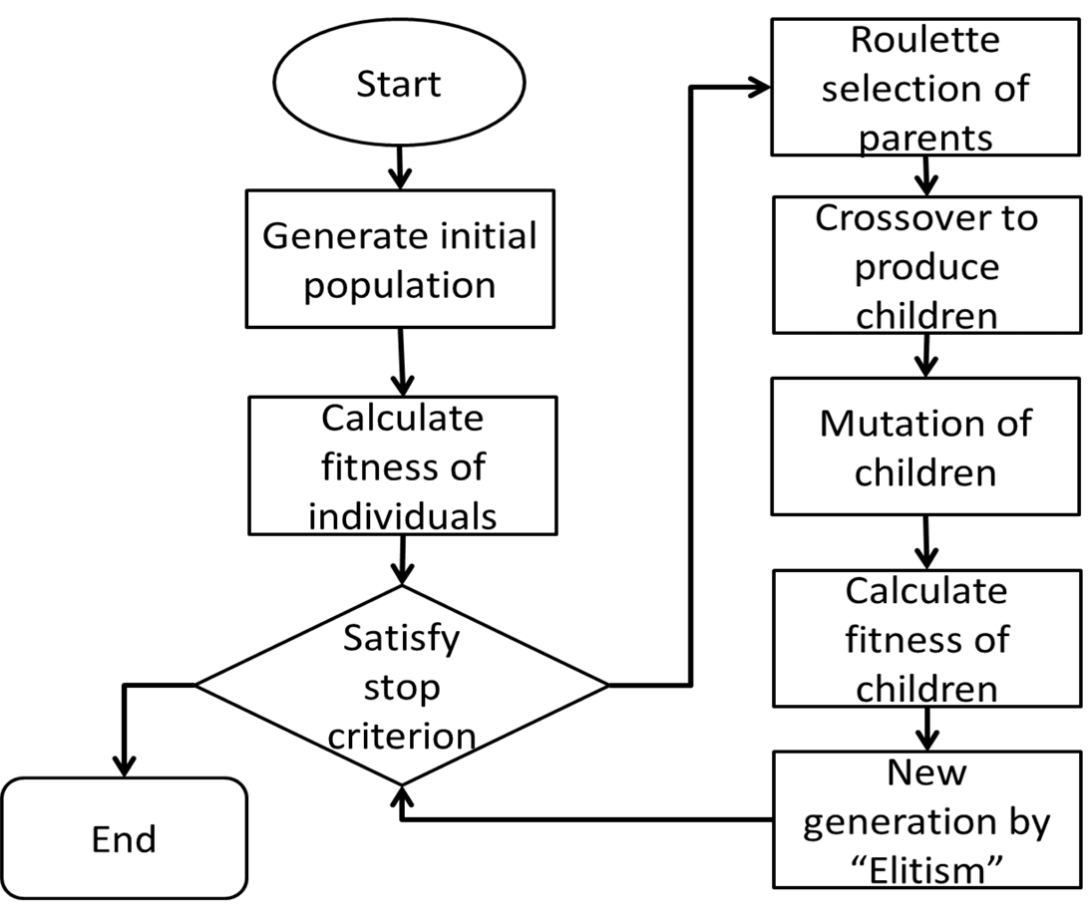
**Flowchart of the Genetic algorithm workflow.** The first population was generated randomly. The fitness criterion is defined as minimizing mean squared error (MSE) between model predictions and experimental results. Roulette wheel selection is used to select candidates for parents in crossover. A fixed mutation rate of 2% was used to prevent premature convergence. The Elitism algorithm was used to determine the survival of parents and children in the new generation

We implemented genetic algorithm to optimize model by MATLAB. Figure 9 shows work flow of the genetic algorithm utilized in this study. We generated first population randomly. The fitness is defined as mean squared error (MSE) between model predictions and experimental results:

The solution will be achieved by minimizing the fitness function through genetic operations. First the parameters in the Boolean transfer function *θ_ij_* and *r_ij_* of the active links are transformed into chromosomal representation. Roulette wheel selection is used to select candidates for parents in crossover. A fixed *mutation rate* of 2% was used to prevent premature convergence. The Elitism algorithm [24] was used to determine the survival of parents and children in the new generation. A population size of 1000 was used. The GA is terminated at 800 generations. Parameter settings of the GA algorithm were in Table 2.

**Table 2:** Settings in genetic algorithm

Chromosome representation	*θ_ij_*,*r_ij_*
Generation number	800
Population size	1000
Crossover rates	1
Mutation rates	0.02

## Authors' contributions

CCK and SCP developed the method, performed the analyses and wrote the manuscript. YJC, CCC and KCT interpreted the data and wrote the manuscript. CYT advised on method design. DSHW investigated the principle and wrote the manuscript.

## Competing interests

The authors declare that they have no competing interests.

## References

[B1] Neil A Campbell JBR Biology 2002 6

[B2] Werner SL Kearns JD Zadorozhnaya V Lynch C O'Dea E Boldin MP Ma A Baltimore D Hoffmann A Encoding NF-kappaB temporal control in response to TNF: distinct roles for the negative regulators IkappaBalpha and A20 Genes Dev 2008 22 2093 2101 10.1101/gad.1680708 18676814 PMC2492747

[B3] Pettigrew MF Resat H Modeling signal transduction networks: a comparison of two stochastic kinetic simulation algorithms J Chem Phys 2005 123 114707 10.1063/1.2018641 16392583

[B4] Mark de Been CF Roy Moezelaar Tjakko Abee Siezen aRJ Comparative analysis of two-component signal transduction systems of Bacillus cereus, Bacillus thuringiensis and Bacillus anthracis Microbiology 2006 152 3035 3048 10.1099/mic.0.29137-0 17005984

[B5] Saez-Rodriguez J Alexopoulos LG Epperlein J Samaga R Lauffenburger DA Klamt S Sorger PK Discrete logic modelling as a means to link protein signalling networks with functional analysis of mammalian signal transduction Mol Syst Biol 2009 5 331 10.1038/msb.2009.87 19953085 PMC2824489

[B6] Julio Saez-Rodriguez LS Jonathan Lindquist A Rebecca Hemenway Ursula Bommhardt Boerge Arndt Utz-Uwe Haus Robert Weismantel Ernst Gilles D Steffen Klamt Burkhart Schraven A Logical Model Provides Insights into T Cell Receptor Signaling PLoS Comput Biol 2007 3 e163 10.1371/journal.pcbi.0030163 17722974 PMC1950951

[B7] Arisi I Cattaneo A Rosato V Parameter estimate of signal transduction pathways BMC Neurosci 2006 7 Suppl 1 S6 10.1186/1471-2202-7-S1-S6 17118160 PMC1775046

[B8] Geiger D Meek C Wexler Y Speeding up HMM algorithms for genetic linkage analysis via chain reductions of the state space Bioinformatics 2009 25 i196 203 10.1093/bioinformatics/btp224 19477987 PMC2687978

[B9] Kauffman SA Metabolic stability and epigenesis in randomly constructed genetic nets Journal of Theoretical Biology 1969 22 437 467 10.1016/0022-5193(69)90015-0 5803332

[B10] Kwon YK Choi SS Cho KH Investigations into the relationship between feedback loops and functional importance of a signal transduction network based on Boolean network modeling BMC Bioinformatics 2007 8 384 10.1186/1471-2105-8-384 17935633 PMC2100072

[B11] Mai Z Liu H Boolean network-based analysis of the apoptosis network: Irreversible apoptosis and stable surviving Journal of Theoretical Biology 2009 259 760 769 10.1016/j.jtbi.2009.04.024 19422837

[B12] Thakar J Pilione M Kirimanjeswara G Harvill ET Albert R Modeling Systems-Level Regulation of Host Immune Responses PLoS Comput Biol 2007 3 e109 10.1371/journal.pcbi.0030109 17559300 PMC1892604

[B13] Holland JH Genetic Algorithms and the Optimal Allocation of Trials SIAM Journal on Computing 1973 2 88 105 10.1137/0202009

[B14] Zheng Y Yeh CW Yang CD Jang SS Chu IM On the local optimal solutions of metabolic regulatory networks using information guided genetic algorithm approach and clustering analysis J Biotechnol 2007 131 159 167 10.1016/j.jbiotec.2007.06.019 17669537

[B15] Wu FX Poirier GG Zhang WJ Inferring gene regulatory networks with time delays using a genetic algorithm Syst Biol (Stevenage) 2005 152 67 74 17044234 10.1049/ip-syb:20050006

[B16] Kanae Oda TK Yukiko Matsuoka Akira Funahashi Masaaki Muramatsu Hiroaki Kitano Molecular Interaction Map of a Macrophage Book Molecular Interaction Map of a Macrophage 2004 2

[B17] Werner SL Barken D Hoffmann A Stimulus Specificity of Gene Expression Programs Determined by Temporal Control of IKK Activity Science 2005 309 1857 1861 10.1126/science.1113319 16166517

[B18] Shih VF-S Kearns JD Basak S Savinova OV Ghosh G Hoffmann A Kinetic control of negative feedback regulators of NF-κB/RelA determines their pathogen- and cytokine-receptor signaling specificity Proc Natl Acad Sci U S A 2009 106 9619 9624 10.1073/pnas.0812367106 19487661 PMC2701028

[B19] Renner F Schmitz ML Autoregulatory feedback loops terminating the NF-kappaB response Trends Biochem Sci 2009 34 128 135 10.1016/j.tibs.2008.12.003 19233657

[B20] Bell E Mediating endotoxin tolerance Nat Rev Immunol 2004 4 750 750 10.1038/nri1463

[B21] Akutsu T Miyano S Kuhara S Identification of genetic networks from a small number of gene expression patterns under the Boolean network model Pac Symp Biocomput 1999 17 28 10380182 10.1142/9789814447300_0003

[B22] Fang-Xiang W Kusalik AJ Wen-Jun Z A genetic algorithm for inferring time delays in gene regulatory networks Computational Systems Bioinformatics Conference, 2004 CSB 2004 Proceedings 2004 IEEE 2004 610 611

[B23] Assmann SM Albert R Discrete dynamic modeling with asynchronous update, or how to model complex systems in the absence of quantitative information Methods Mol Biol 2009 553 207 225 full_text 19588107 10.1007/978-1-60327-563-7_10

[B24] Grefenstette J Optimization of control parameters for genetic algorithms IEEE Trans Syst Man Cybern 1986 16 1 122 128 10.1109/TSMC.1986.289288

